# Zhenwu decoction for chronic heart failure: Protocol for a systematic review and meta-analysis

**DOI:** 10.1097/MD.0000000000011559

**Published:** 2018-07-20

**Authors:** Qi Tang, Yuanping Wang, Kuangyi Li

**Affiliations:** a Guangzhou University of Chinese Medicine, Guangzhou; b Department of Emergency, Foshan Hospital of TCM, Affiliated Hospital of Guangzhou University of Chinese Medicine, Foshan, China.

**Keywords:** chronic heart failure, protocol, systematic review, Zhenwu decoration

## Abstract

**Background:**

Chronic heart failure (CHF), not only a severe personal healthy problem but also a social financial burden, has become a global health concern. Zhenwu decoction (ZWD) has been widely adopted to clinical practice for patients with CHF. It is necessary to update the researches to reevaluate the efficacy and safety of ZWD to provide the up-to-date evidence for CHF management. Therefore, we provide a protocol of systematic review regarding ZWD for CHF.

**Methods:**

The aim of this study was to retrieve papers on the topic of ZWD treatment for CHF in the electrical databases, including PubMed, Cochrane Library, EMBASE, Chinese Biomedical database (CBM), Chinese National Knowledge Infrastructure (CNKI), Wanfang database, and Chinese Science and Technology Periodical database (VIP). The included literatures are those delivered from the time when the databases were established to May 2018. The therapeutic effects based on the mortality and the New York Heart Association (NYHA) function classification will be marked as the primary outcomes. We will use RevMan V.5.3 software to calculate the data synthesis as well as to perform meta-analysis if the results are appropriate.

**Results:**

The literature will provide a high-quality synthesis of current evidence of ZWD for CHF from various comprehensive assessment, including mortality, NYHA function classification, left ventricular ejection fraction, 6-minute walk test (6MWT), life quality, and adverse events.

**Conclusion:**

The systematic review will provide up-to-date evidence to assess ZWD treatment effect for patient with CHF.

**PROSPERO registration number:**

PROSPERO CRD42018083813.

## Abstract 2

Supplemental Digital Content is available in the text

## 1 Introduction

With a series of complained symptoms (breathlessness, orthopnea, paroxysmal nocturnal dyspnea, reduced exercise tolerance, fatigue, and ankle swelling), chronic heart failure (CHF) has become a major global health issue characterized as an abnormality of damage of ventricular filling or the ejection of blood, resulting in hypoxia of body organs.^[1]^ Incidence of CHF rises year by year, because the worldwide epidemic increases with the general aging of the population. American Heart Association (AHA) investigated that more than 37.7 million individuals globally had diagnosed CHF clearly, a figure sure to have risen since it was presented in 2012.^[2]^ In-hospital heart failure (HHF) is the leading cause of hospitalization in the United States and Europe, resulting in more than 1 million hospital admissions as the primary diagnosis, accounting for 1% to 2% of all hospitalizations.^[3]^ Patients with CHF suffer from poor quality of life and social activities for the unpredictable risk of deterioration, repeated symptoms, high hospitalization and readmission rates, anxiety and depression emotion, and poor prognosis, even death. ^[4,5]^ With high hospitalization rates, the health care cost of CHF is a heavy financial burden either for families or countries around the globe.^[6]^ Data from AHA stated that the total medical costs are estimated to increase from US$20.9 billion in 2012 to $53.1 billion by 2030 in the USA.^[7]^ In the meantime, developing countries also put the cost of CHF at US$15.1 billion.^[6]^

The goal of treatment management for CHF patients is to prevent myocardial remodeling to gain improvement of clinical status, functional capacity, and quality of life, and reduce mortality. Currently, standard western medicines recommended to improve cardiac function for CHF are angiotensin-converting enzyme inhibitor (ACEI), b-blocker, aldosterone inhibitor, digoxin, and diuretics.^[8]^ However, these strategies cannot obtain a desired satisfaction, owing to the poor physical function, lower heart rate, and side effects of the pharmacological treatment. As result, it is essential to look for a method treating CHF effectively with less side effects.

Chinese herbal medicine (CHM), used as a part of complementary and alternative medicine (CAM) originating from ancient China, has been used to treat heart diseases CHF for thousands of years in China. In the last decades, traditional herbal prescription and patent herbal products coexist in the treatment of CHF.^[9]^ Zhenwu decoction (ZWD), as a well-known CHM in CHF treatment in China, is composed of Cinnamomi Ramlus, Zingibers Rhizoma Recens, Poria, Aconiti Lateralis Radix Praeparata, and Atractylodis Macrocephalae Rhizoma. Numbers of researches and systematic reviews reported that ZWD had more significant improvement than western medicine conventional therapy in left ventricular ejection fraction (LVEF) and clinical symptoms for patients with CHF.^[10]^

An animal experimental study demonstrated that ZWD delayed the progression of CHF in rats and increased myocardial contractility in CHF rats to improve the symptoms of heart failure significantly by the means of regulating the levels of sFas and sFas-L in serum, and also affected the apoptosis of myocardial cells in CHF rats.^[11]^ Another experiment found that ZWD improved cardiac function in CHF rats by reducing Bax expression in cardiomyocytes and counterpoising Bcl-2 and Bax.^[12]^

According to the best of our knowledge, we found only 2 prior systematic reviews^[10,13]^ demonstrating the efficacy regarding ZWD in CHF. However, both the reviews have a limitation in providing rigorous medical evidence owing to the low quality and small sample of the included studies. Therefore, we are aiming to update a systematic review to identify the efficacy and safety of ZWD for CHF with high-quality and large sample evidence.

## 2 Methods

### 2.1 Inclusion criteria for study selection

#### 2.1.1 Types of studies

All the relevant randomized controlled trials (RCTs) regarding ZWT for the treatment of CHF will be included. Quasi-randomized and observational studies will be excluded. No language or publication status constraints will be placed.

#### 2.1.2 Types of patients

Participants, adult patients (18 years of age and older) who are clinically diagnosed with CHF, according to the Guidelines on the Diagnosis and Treatment of Heart Failure,^[8]^ will be included. Gender and race will not be considered.

#### 2.1.3 Types of interventions

The intervention in eligible researches of interest is the experimental group, which will be treated with western medications (WMs) and CHF, while the control group will receive the same WM (e.g., ACEI, b-blocker, aldosterone inhibitor).

#### 2.1.4 Types of outcome measures

##### 2.1.4.1 Major outcomes

**The major outcomes included**
• Mortality;
• Clinical total effective rate;
• NYHA function classification.

##### 2.1.4.2 Secondary outcomes

**The secondary outcomes included**
• Quality of life as measured by various instruments;
• LVEF;
• Exercise test or 6-minute walk test performance (6MWT);
• Hospitalization and rehospitalization;
• Adverse effects.

### 2.2 Search methods for the identification of studies

#### 2.2.1 Electronic searches

EMBASE, PubMed, the Cochrane Central Register of Controlled Trials (Cochrane Library), and Medline 4 English databases and 3 Chinese databases Chinese Biomedical Literature Database (CBM), China National Knowledge Infrastructure (CNKI), and Chinese Science and Technology Periodical database (VIP) will be searched from inception to May 2018 for the relevant RCTs of ZWD for CHF. On the basis of the Cochrane handbook, detailed strategies for searching the PubMed database in Appendix A, and similar strategies will be applied to remaining databases.

#### 2.2.2 Searching other resources

We will manually review the references lists of included studies and previous systematic reviews for other potentially relevant literatures. Also, conference proceedings for AHA, American College of Cardiology (ACC), and European Society of Cardiology (ESC) meetings will be retrieved in the last 5 years. What is more, we will contact experts in the field to see if they are aware of other studies on the topic.

### 2.3 Data collection and analysis

#### 2.3.1 Selection of studies

**Figure 1:**
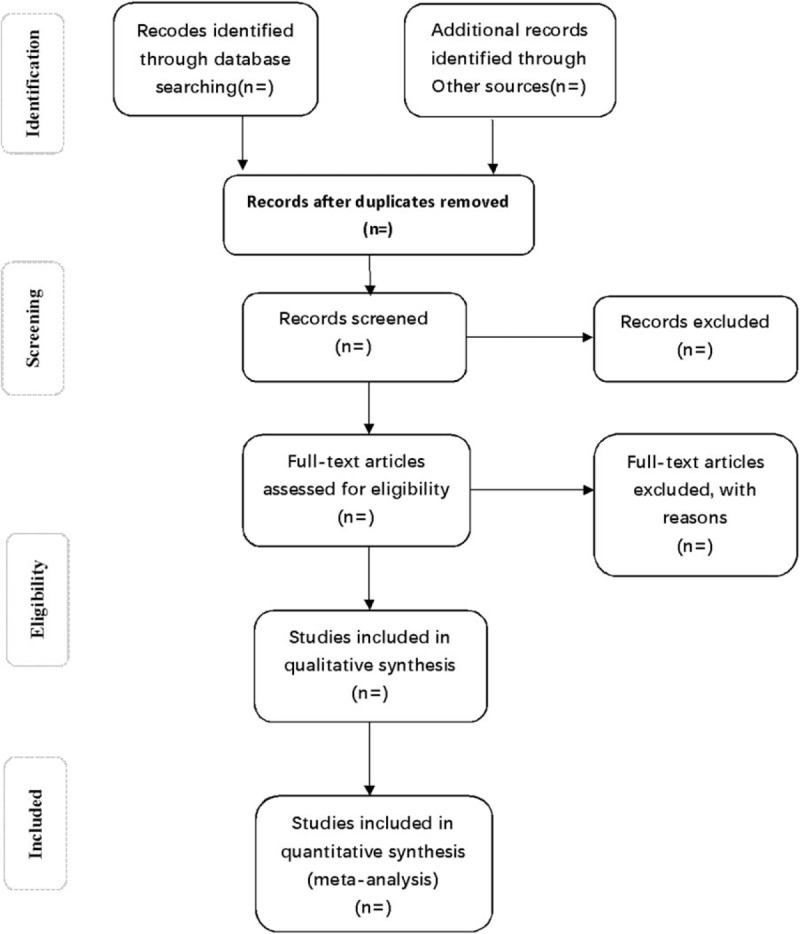
Flow diagram of study selection process.

Two experienced authors will first search all databases independently to screen the titles and abstracts of relevant studies removing duplication. Another 2 independent researchers will review all the extracted titles and abstracts, and screen full texts based on the previous inclusion criteria to identify eligible studies. Studies removed after full text review will be recorded with specific exclusion reason. Any disagreement will be solved by group discussion. If necessary, the third reviewer (c) will be consulted. The selection process of eligible papers is shown in a Preferred Reporting Items for Systematic Review and Meta-analysis (PRISMA) flow diagram (Fig. 1).

#### 2.3.2 Data collection and management

After identification of all included studies, the same 2 reviewers will conduct data collection independently using prediluted extraction forms. Data extraction will include study characteristics, population characteristics, follow-up assessment method, details of the treatment and control group, as well as relevant indicators of bias risk assessment. In the event that above-mentioned data are not reported in the primary study, we will contact the corresponding author of the primary study for additional information. Any divided opinions between reviewers that cannot be resolved through discussion will be referred to a third reviewer.

#### 2.3.3 Assessment of risk of bias in included studies

Cochrane Collaboration tool^[14]^ will be adopted to the assessment of risk of bias. Two researchers will independently be in charge of evaluation for each included study. Each original research will be assessed in 6 domains: random sequence generation, allocation concealment, blinding of participants and personnel, blinding of outcome assessment, selective reporting and other sources of bias, and classified as “low,” “high,” or “unclear” according to the tool. If there is any disagreement in procession, we will reach a consensus through discussion or consult with a senior author.

#### 2.3.4 Measures of treatment effect

Using RevMan 5.3 [Review Manager (RevMan) (Computer program), Version 5.3, Copenhagen: The Nordic Cochrane Center, The Cochrane Collaboration, 2014), we will perform a meta-analysis if the results are appropriate. For dichotomous outcomes, the extracted data will be presented as rate ratio (RR) with 95% confidence interval (95% CI). As for continuous data, results will be computed as mean difference (MD) with 95% CI.

#### 2.3.5 Dealing with missing data

If required information is not available in included literatures, the authors will connect with the corresponding author of the primary studies by E-mail for complete information. If no additional message is received, we will conduct data synthesis using available data. But at the same time, we will also discuss the possible consequence the missing data might cause in the review.

#### 2.3.6 Assessment of heterogeneity

Heterogeneity among trials will be undertaken to evaluate the feasibility of meta-analysis. If *I*^2^ value is over 50%, we will consider the significant heterogeneity and perform subgroup analysis to investigate the potential causes from clinical or methodological heterogeneity.

#### 2.3.7 Assessment of reporting bias

If figure of included trials is adequate (over 10 pieces) in the review, we will put funnel plot according to Egger methods to discuss the reporting biases or small-study effects.

#### 2.3.8 Data synthesis

RevMan software will be computed to calculate for data synthesis when a meta-analysis is suitable. On the condition that no obvious statistical heterogeneity is found among the trails, fixed effects model will be performed in the analysis. If not, the reviewers will detect the source of statistical heterogeneity in further analysis. However, if apparent clinical heterogeneity is exploded, then the random effects model will be employed. At the same time, subgroup or sensitivity analysis will be carried out. α = 0.05 will be deemed statistical significant. If it is not available to conduct a meta-analysis, we will only describe the data.

#### 2.3.9 Subgroup analysis

Subgroup analysis will be carried out in the premise of sufficient eligible studies (at least 10 trials). Exploring the resources of the heterogeneity, we will assess inconsistent participants characteristic, classification of CHF, disease course, dose of taking medicine, and other unpredictable factors.

#### 2.3.10 Sensitivity analysis

Sensitivity analysis will be adopted to detect the quality of included studies of the document following sample size, the outcome of missing data, and methodological quality.

#### 2.3.11 Ethics and dissemination

The results of the systematic review will be disseminated via publication in a peer-reviewed journal and presented at a relevant conference. The data we will use do not include individual patient data, so ethical approval is not required.

## 3 Discussion

CHF, with a large aging population, is a global epidemic in health care during the past decades.^[15]^ Numerous researches have demonstrated that ZWD is an effective treatment method for CHF. There have been 2 systematic reviews^[10,13]^ that investigated the effectiveness and safety of ZWD on CHF. However, due to the low quality of the included literature. We have displayed a protocol for a systematic review providing up-to-date data to detect the effectiveness and safety of ZWD for CHF. It cannot be denied that plenty of clinical studies on this topic were published, although a high-quality trial is still lacking; therefore, we begin to conduct the review when necessary trails are meeting and all operating procedures will be performed in accordance of Cochrane Handbook to ensure the provided helpful information for clinicians and CHF patients.

## Author contributions

**Conceptualization:** Qi Tang.

**Data curation:** Yuanping Wang.

**Formal analysis:** Yuanping Wang, Kuangyi Li.

**Funding acquisition:** Kuangyi Li.

## Supplementary Material

Supplemental Digital Content
