# Supplementary material for: Zhenwu decoction for chronic heart failure: Protocol for a systematic review and meta-analysis
Source: Medicine (Baltimore). 2018 Jul 20;97(29):e11559. doi: 10.1097/MD.0000000000011559 (PMC6086473; doi:10.1097/MD.0000000000011559)
Supplement: Supplemental Digital Content [file medi-97-e11559-s001.docx]

**Appendix A.**

***Search strategy used in PubMed database***

#1 Zhenwutang OR Zhenwu Decoction

#2 Heart Failure OR Cardiac Failure OR Heart Decompensation OR Decompensation, Heart OR Heart Failure, Right-Sided OR Heart Failure, Right Sided OR Right-Sided Heart Failure OR Right Sided Heart Failure OR Myocardial Failure OR Congestive Heart Failure OR Heart Failure, Congestive OR Heart Failure, Left-Sided OR Heart Failure, Left Sided OR Left-Sided Heart Failure OR Left Sided Heart Failure

#3 Randomized controlled trial OR clinical study OR Clinical Trial OR Controlled study OR Controlled Trial OR Random*Control* study OR random* Control* Trial

#1 AND #2 AND #3
